# Crystallographic phase retrieval method for liquid crystal bicontinuous phases: indicator-based method

**DOI:** 10.1107/S2053273322006970

**Published:** 2022-07-28

**Authors:** Toshihiko Oka

**Affiliations:** a Department of Physics, Faculty of Science, Shizuoka University, Shizuoka, 422-8529, Japan; b Nanomaterials Research Division, Research Institute of Electronics, Shizuoka University, Shizuoka, 422-8529, Japan; University of Patras, Greece

**Keywords:** lyotropic liquid crystals, triply periodic minimal surfaces, crystallographic phase retrieval

## Abstract

An indicator-based crystallographic phase retrieval method has been developed for diffraction data of bicontinuous cubic phases of lyotropic liquid crystals. Such liquid crystals have large structural disorder; the number of independent Bragg reflections that can be observed is limited. This paper proposes two indicators to identify plausible combination(s) of crystallographic phases, *i.e.* electron-density distribution. The indicators are based on the characteristics of the liquid crystals: amphiphilic molecules diffuse mainly in the direction parallel to polar–nonpolar interfaces and the electron density in the direction parallel to the interfaces is averaged. One indicator is the difference between the maximum and minimum electron density, and the other is calculated from the Hessian matrix of the electron density. Using test data, the electron densities were calculated for all possible phase combinations and indicators were obtained. The results indicated that the electron densities with the minimum indicators were close to the true electron density. Therefore, this method is effective for phase retrieval. The accuracy of the phase retrieval decreased when the volume fraction of the region including the triply periodic minimal surface increased.

## Abstract 2

An indicator-based crystallographic phase retrieval method has been developed for diffraction data of bicontinuous cubic phases of lyotropic liquid crystals. The electron densities with the minimum indicators are close to the true electron density.

## 1. Introduction

Restoring the phases of the structure factors is a crucial problem in crystal structure determination from X-ray diffraction (XRD) data. Direct methods solve the phase problem by combining the intensity information with constraints based on the expected structural information (atomicity, positivity and zero density regions) (Sayre, 1952 ▸; Giacovazzo, 2001 ▸). Closely related is the charge-flipping method, an efficient iterative phase retrieval method with similar requirements that has become popular in recent years (Oszlányi & Sütő, 2004 ▸, 2008 ▸). It may be possible to obtain the structure using data from samples that satisfy the structural information; however, this method is limited to solid crystals in which the atomic positions are almost fixed.

**Figure 1:**
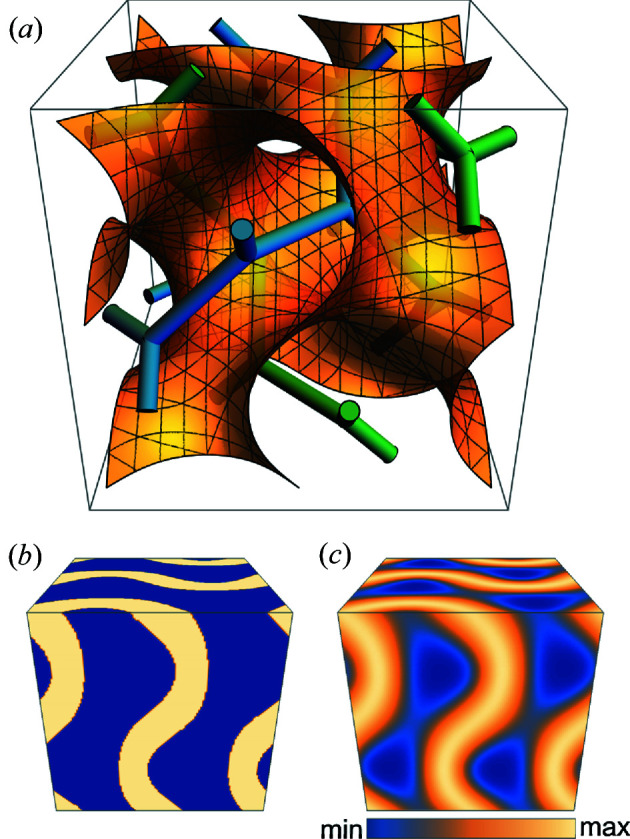
(*a*) A gyroid (G) surface (orange), one of the triply periodic minimal surfaces (TPMSs), and the networks (green and blue) through the centers of the two interwoven spaces where the surface divides. A single unit cell is shown. (*b*) Two regions separated by a surface (an interface) of constant thickness from the TPMS: TPMS side (yellow) and network side (blue). The volume fraction of the TPMS side is 0.4. (*c*) Structural model with fluctuations. In the structure shown in (*b*), the TPMS side region is set to an electron density of 1 and the network side region is set to 0, and a Gaussian function is convolved as a fluctuation.

Liquid crystals have properties that are intermediate between solids and liquids. In liquid crystals, the molecules move like a liquid in a certain direction, but are oriented like a solid crystal in other directions. For the latter reason, many liquid crystal phases have periodic structures and are subject to XRD measurements. Bicontinuous cubic phases of lyotropic liquid crystals (LLCs) have structures similar to triply periodic minimal surfaces (TPMSs) (Hyde *et al.*, 1996 ▸), and diffraction data can be obtained as if they were 3D crystals [Fig. 1 ▸(*a*)]. However, the spatial resolution of the observed XRD data is low due to structural disorder, and the number of independent reflections is small. The previously mentioned constraints, except the positivity, are not satisfied. There is thus a problem in restoring the electron density from XRD data, although the centrosymmetry of the LLC bicontinuous phase restricts the phase to 0 or π. Luzzatti and colleagues first clarified the electron-density distribution of an LLC bicontinuous phase (Luzzati *et al.*, 1988 ▸; Mariani *et al.*, 1988 ▸). They proposed to use the average of the fourth moment of the electron density as an indicator for phase retrieval. They insisted that the combination of the phases at the smallest value was the most reliable. However, as they admitted, the electron density with the minimum indicator was not always the proper phase combination. Other methods have also been proposed for LLCs, such as looking for the position of the methyl group in the hydro­carbon chain of a lipid bilayer (Harper *et al.*, 2000 ▸).

We have performed X-ray crystallographic studies of LLC bicontinuous cubic phases using single-crystal regions of samples (Oka, 2017 ▸; Oka *et al.*, 2018 ▸, 2020 ▸). In these studies, the structures of the bicontinuous cubic phases were determined using models based on available information. However, it would be better if the structure of the bicontinuous cubic phase could be revealed without a structural model. Here, a simple indicator-based phase retrieval method is reported. Two valuable indicators have been identified based on the universal characteristics of the LLC bicontinuous structures. The phase combination in which these indicators are minimized is very close to the true phase combination, and makes it possible to retrieve the crystallographic phase of the TPMS-like structure. Although this method was designed to clarify the structure of the LLC bicontinuous cubic phases, it would be applicable to thermotropic liquid crystals, polymers, and other materials with TPMS-like structures.

## 2. Indicators for bicontinuous structure (TPMS-like structure)

### 2.1. Bicontinuous structure (TPMS-like structure)

**Figure 2:**
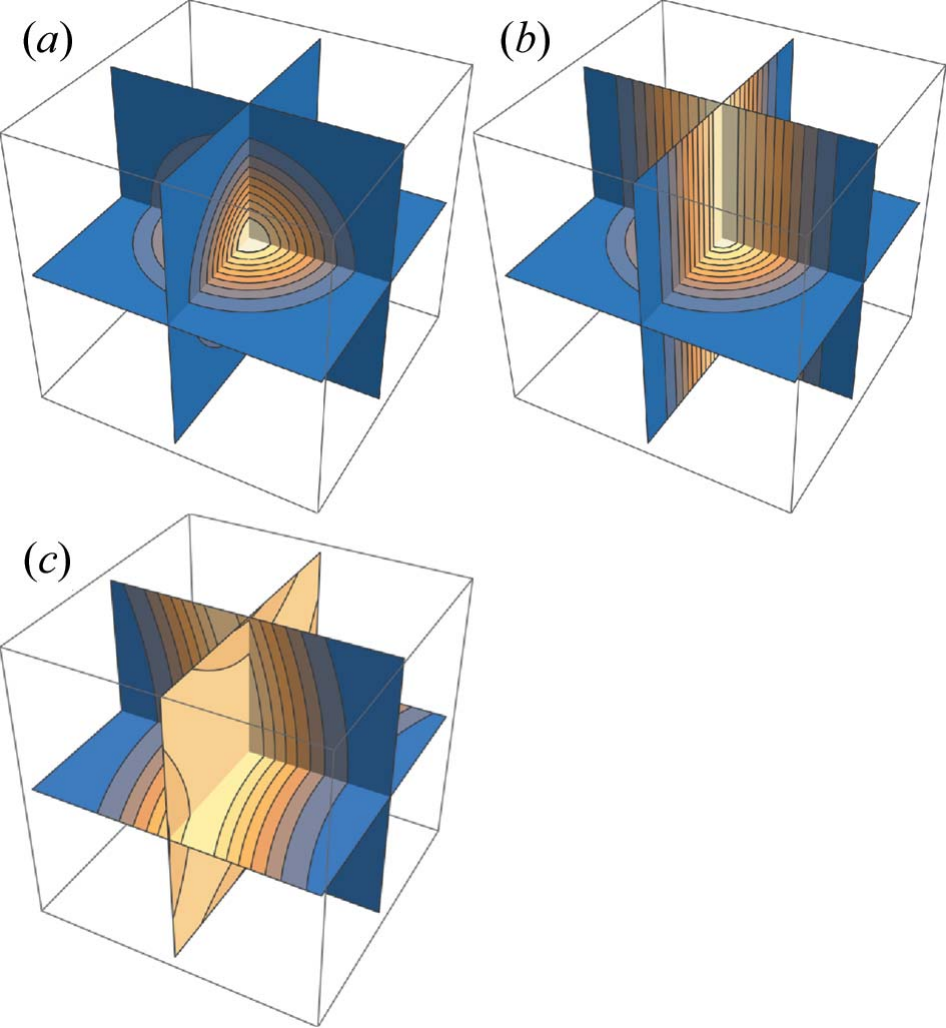
Sliced contour plots of idealized electron densities of (*a*) sphere-like, (*b*) cylinder-like and (*c*) minimal surface like structures. Only in (*a*) is there a strictly convex upward density region where all eigenvalues of the Hessian matrix are negative. The strictly convex upward density region forms a closed isoelectron density surface. On the other hand, there is no closed isoelectron density surface in (*b*) and (*c*).

An LLC consists of two or more components, amphiphilic molecules and water (or oil). There are two types of LLC, oil-in-water and water-in-oil types, commonly referred to as type I and type II, respectively (Israelachvili, 2011 ▸). In type II bicontinuous cubic phases of LLCs, three different TPMS-like structures, P surface, D surface and gyroid (G) surface, have been observed, while only the G surface has been observed in type I (Hyde *et al.*, 1996 ▸). The space groups of the three TPMS-like structures of P, D and G are 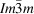 , 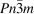 and 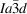 , respectively. In type II, the nonpolar region contains the TPMS as the center surface, and the polar region is located on the two networks separated by the TPMS [Fig. 1 ▸(*a*)]. In the type I bicontinuous cubic phase, the positions of the polar and nonpolar regions are swapped with those of type II (Israelachvili, 2011 ▸). In both cases, the polar–nonpolar interface is on the amphiphilic molecule, and the amphiphilic molecule can move like a liquid molecule in the direction parallel to the polar–nonpolar interface. Therefore, the electron density in the direction parallel to the interface is averaged, but not that in the perpendicular direction. This averaging leads to the separation of the polar and nonpolar regions. In the case of an amphiphilic molecule and water, if the electron densities of the polar parts of the amphiphilic molecules and waters are almost the same, then the electron density can be described in two levels, polar and nonpolar, without considering fluctuations. The region in the TPMS is a minimal surface like structure, and the regions on the networks are cylinder-like structures with branches (Fig. 2 ▸). If the electron densities of the polar parts of water and amphiphiles are different, then the electron density can be described in three continuous regions, located on or along the TPMS or the networks.

### 2.2. Indicator *I*_ρ_

The bicontinuous structure consists of two (or three) flat electron-density regions if fluctuations are not considered [Fig. 1 ▸(*b*)]. If electron density is restored using structure factors with incorrect phases, then the density in the flat region will undulate and the difference between the minimum and maximum electron densities will become more significant compared with the true density. Therefore, the difference between the minimum electron density in a unit cell, 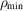 , and the maximum electron density, 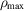 , would serve as an indicator:

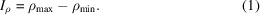

This indicator corresponds to the range in statistics. A smaller indicator is a better candidate for the proper phase solution. The actual electron density is close to the step-like electron density with the convolution of the fluctuation function [Fig. 1 ▸(*c*)]. In this case, the electron density is almost constant at a maximum or minimum on the TPMS and the networks [Fig. 1 ▸(*a*)]. Electron densities calculated with incorrect phases will undulate on the TPMS or networks, which increases the indicator 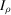 .

### 2.3. Indicator *I_K_*

A minimal surface like structure located on the TPMS and cylinder-like structures with junctions located on the networks are close to parts of the electron density of the LLC bicontinuous cubic phases [Figs. 2 ▸(*b*) and 2 ▸(*c*)]. The molecules diffuse along the structural motifs; therefore, the isoelectron density surfaces are not closed but infinitely connected. However, only the sphere-like structure in Fig. 2 ▸ has closed isoelectron density surfaces, *i.e.* a region that is strictly convex upward. An isolated density maximum/minimum is difficult to imagine in the LLC bicontinuous cubic phase because it seemingly contradicts molecular diffusion over a long distance. Therefore, few density regions that are convex upward or downward are expected. Convex regions can be determined by the eigenvalues of the Hessian matrix of the electron density, 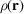 :

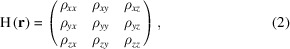

where subscripts indicate partial derivatives. If all eigenvalues of the Hessian matrix are positive, then the region is strictly convex downward, and if all are negative, then the region is strictly convex upward (Rockafellar & Wets, 2010 ▸). Let *C* be the electron-density regions that are convex upward or downward and let *K*(**r**) be the determinant of H(**r**): *K*(**r**) = det[H(**r**)]. Therefore, *K*(**r**) is the product of three eigenvalues. The following indicator was considered:

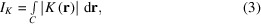

which is the integral of the absolute value of 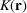 in the region *C* of the electron density in which the three eigenvalues are of the same sign in a unit cell, where **r** is the position in a unit cell. Thus, *I_K_* is an indicator that expresses the total (integrated) convexity. With regard to the geometrical meaning, the eigenvalues of the Hessian matrix are the principal curvatures, and *K*(**r**) is the Gaussian curvature if the 3D electron density is regarded as a hypersurface in a 4D hyperspace (Monga & Benayoun, 1995 ▸; Goldman, 2005 ▸).

## 3. Phase retrieval method based on the *I*_ρ_ and *I*_*K*_ indicators

The structure factor 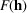 can be divided into the amplitude 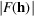 and the phase part 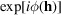 , where 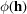 is the phase. In a diffraction experiment, intensities are measured as the squares of the amplitudes and the phases are not observable. The electron density 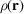 can be obtained by Fourier transformation of the structure factors as follows:

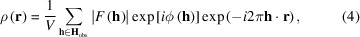

where **r** is the position in the unit cell, **h** is the reciprocal-lattice vector and *V* is the volume of the unit cell. **H**_obs_ is the set of **h** for which the structure factor could be observed. *F*(000) was not used in this paper; therefore, the integrated value of the electron density 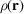 in the unit cell is zero. High-electron-density regions are thus positive, whereas low-electron-density regions are expressed as negative in 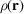 .

In the phase retrieval, the electron densities were calculated for all possible phase combinations by placing them into the phase part 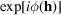 of the structure factor. The structures of the bicontinuous cubic phases are centrosymmetric, so that the phase values are 0 or π (if the unit-cell origin is placed on a symmetry center), *i.e.* the structure factors are real (positive or negative, respectively). When *n* is the number of the independent structure-factor amplitude, the corresponding number of phase combinations is 2*^n^*. The electron density was calculated in 32 × 32 × 32 voxels as a unit cell. 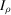 was obtained from the difference between the maximum and minimum electron densities. To obtain *I_K_*, the Hessian matrices were obtained from the electron densities for all voxel points in the unit cell, which yielded three eigenvalues at all points. The region where the signs of the three eigenvalues were the same was designated as *C*, and *K*(**r**) was obtained at each point within *C*. *I*_*K*_ was then calculated by integration as described in Section 2.3. The fourth moment of the electron density 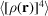 was calculated at the same time (Luzzati *et al.*, 1988 ▸). *F*(000) was not used in this paper; therefore, 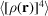 corresponds to the average of the fourth power of the electron density, and 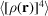 was calculated as the average of the fourth power of the electron density 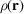 in the unit cell.

According to the Babinet principle, when the electron density 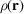 is inverted, the amplitudes of the structure factors are the same, but the phases are shifted by π. Therefore, the structures with inverted electron densities were considered equivalent, and the number of phase combinations to be calculated was reduced. In the space group 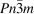 , if the origin of a structure is (0,0,0), then moving the origin to (1/2,1/2,1/2) will give equivalent amplitudes of the structure factors, although some phases are shifted by π (Giacovazzo, 2001 ▸). Therefore, it was considered that the structure is equivalent, even when the translation operation of (1/2,1/2,1/2) is performed, and the number of phase combinations to be calculated is reduced. The electron densities and indicators were calculated using these structure factors. Phase retrieval was conducted using an in-house-made Python3 script.

## 4. Test data and quality of phase retrieval

### 4.1. Test data derived from constructed models

Electron-density models were constructed to test the indicators for the phase retrieval. Two polar–nonpolar interface models for the LLC bicontinuous cubic phase were used: a parallel surface (PS) (Hyde *et al.*, 1996 ▸) and a constant mean curvature surface (CMCS) (Anderson *et al.*, 1990 ▸; Große-Brauckmann, 1997 ▸). In the PS model, the polar–nonpolar interface is parallel to the TPMS, and in the CMCS model, the interface is a CMCS. The PS and CMCS models were generated as model electron densities from the three TPMSs, *i.e.* the G, P and D surfaces. The PS and CMCS models based on the G surface are referred to as the G-PS and G-CMCS models, respectively.

The TPMSs and CMCSs were created using *Surface Evolver* (Brakke, 1992 ▸; Shearman *et al.*, 2007 ▸). The PSs were generated as surfaces at fixed distances from the TPMSs [Fig. 1 ▸(*b*)]. Stepwise electron densities were created for both models. The electron density was calculated in 128 × 128 × 128 voxels as a unit cell. The density from the interface to the TPMS side was set to 1.0, and the rest of the region was set to 0.0. The volume fractions from the interface to the TPMS side ( 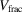 ) were 0.2, 0.4, 0.6, 0.7 and 0.8. PS models with different 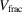 were generated by varying the constant distance from the TPMS to the polar–nonpolar interface (Qiu & Caffrey, 1998 ▸), while the CMCS model requires only a volume fraction to generate the CMCS. The CMCS with 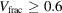 could not be generated for the P surface; therefore, only P-CMCS models with 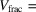 0.2 and 0.4 were used. Using values from previous papers as a reference (Oka, 2017 ▸; Oka *et al.*, 2018 ▸, 2020 ▸), the lattice constant was set to 1 and the width of the Gaussian function was set to 0.05 for the G surface based models and 0.07 for the other models.

The structure factors 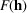 were obtained by Fourier transformation of the model densities 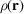 :

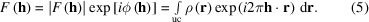

Integration is performed in a unit cell. The numbers of independent structure-factor amplitudes used were 22, 19 and 13 for the G, D and P surface based models, respectively. The model density construction, except for the formation of the TPMS and CMCS, and other calculations were conducted using *Mathematica 12.1* (Wolfram Research, Inc., USA).

### 4.2. *R*_p_ value to evaluate the quality of phase retrieval

The *R*_p_ value is defined as a quantity that evaluates how well the phase combination used in the calculation agrees with the true phase combination:

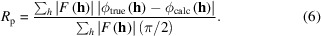


*R*_p_ close to 0 indicates a good agreement with the true phase combination. From the Babinet principle, the inverted electron density was also treated in the same way, and the π-shifted phase combination from 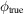 was also treated as the true phase. Of the *R*_p_ calculated from the original and π-shifted phase combinations, that with the smaller value was adopted.

## 5. Results

### 5.1. Phase retrieval for constructed models

Phase retrieval was performed for model densities with known true phase combinations. The electron density was calculated for all phase combinations, and the corresponding 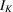 and 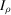 indicators were calculated.

**Figure 3:**
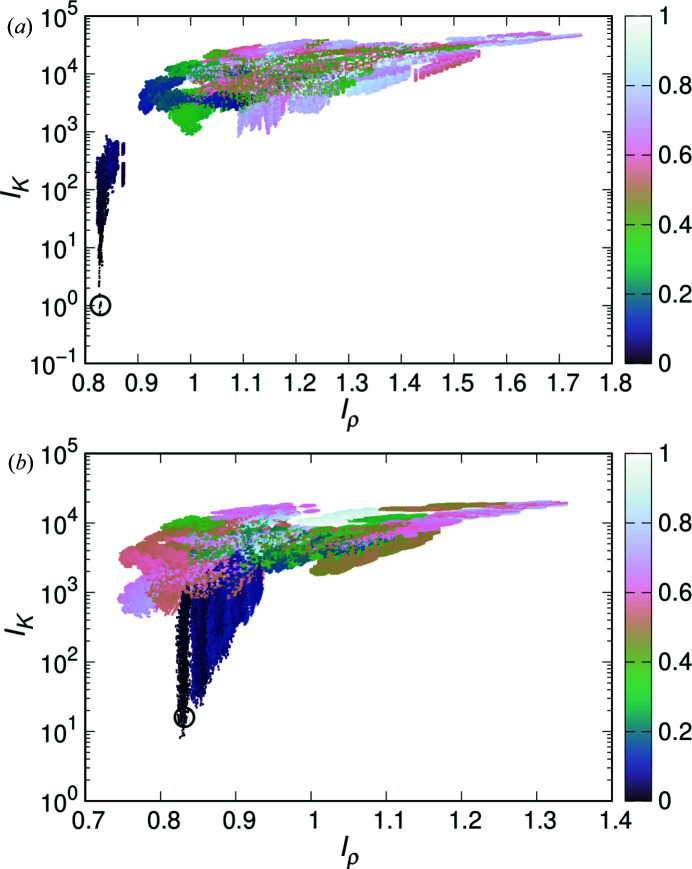
Distributions of the 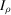 and 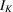 indicators of G-PS models at (*a*) 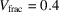 and (*b*) 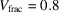 . The indicators were calculated from the electron densities using all phase combinations. The colors of the points indicate *R*_p_ that compare the phase combinations with the true phase combination. The black circles show the true phase combination indicators of the model.

Fig. 3 ▸(*a*) shows the distribution of 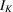 and 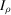 for all phase combinations at 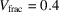 of the G-PS model. The points are distributed from the lower left to upper right. Therefore, there is an approximate positive correlation between 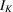 and 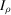 . The *R*_p_ values of the points tend to be smaller in the lower left region; points with *R*_p_ of approximately 0.1 or less are concentrated in the lower left region. 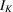 and 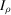 for the true phase combination are in the lower left corner, which indicates that both indicators of the true phase are almost the smallest, as expected; the same is true when 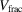 is 0.2 and 0.6 in the other models (Fig. S1 in the supporting information). However, when 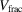 is 0.8, 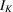 for the true phase combination is roughly minimized, whereas 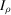 is clearly not minimized [Fig. 3 ▸(*b*)]. The tendency that the indicator for the true phase combination is not minimized when 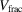 is large is also true for the G-CMCS model and the other surface-based models (Fig. S1). For example, in the G-CMCS model, both 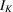 and 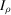 are not minimized when 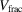 is 0.7 or 0.8.

**Figure 4:**
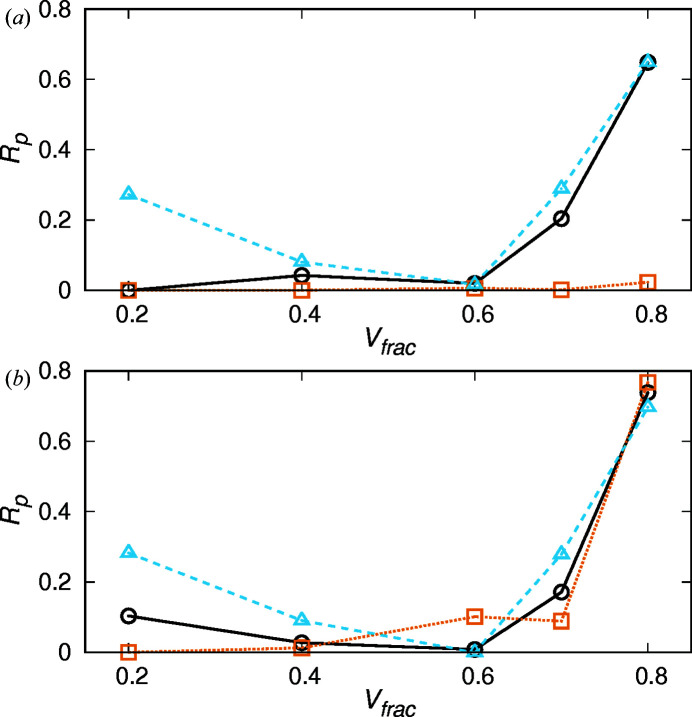
*R*_p_ of the phase combinations with the minimum indicators in models based on (*a*) G-PS and (*b*) G-CMCS surfaces: 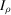 (black circles) and 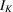 (orange squares), and 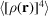 (sky-blue triangles).

The *R*_p_ values of the phase combinations with the minimum indicators in the G-PS and G-CMCS models are shown in Fig. 4 ▸. When the volume fraction is smaller than 0.6, *R*_p_ of the phase combinations at the minimum indicators is approximately 0.1 or less. Therefore, in this range of volume fractions, both 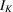 and 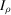 indicators are useful for phase retrieval. When *V*_frac_ is 0.7 or 0.8, some of the phase combinations at the minimum indicator are *R*_p_ > 0.2, which indicates that the phase retrieval by the indicator is not realized. In particular, the phase combination with the minimum 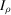 differs significantly from the true combination for 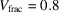 with all the models. This indicates that when *V*_frac_ is large, the constraint that the difference between the minimum and maximum electron density is minimal does not hold. On the other hand, the *R*_p_ value for minimum 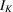 is relatively small, even when the volume fraction is 0.7 or 0.8. Including other models, *R*_p_ > 0.2 with minimum 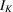 is limited to three conditions: the G-CMCS model with 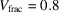 and the P-PS model with 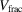 = 0.7 and 0.8. Therefore, 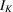 is useful for phase retrieval to some extent, even if 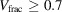 . When *V*_frac_ is large in the G-CMCS model, the interface structure deviates from the simple model described in Section 2 due to the junctions of the networks (Große-Brauckmann, 1997 ▸). In the P-PS model, the junction has six branches, so if the 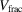 is large, the junction is far outside the cylinder structure. The combination of these factors and the smearing caused by fluctuations leads to a failure in the phase retrieval of 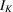 because the electron density is out of the constraint based on the eigenvalues.

The previously used indicator, the fourth moment of the electron density 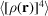 (Luzzati *et al.*, 1988 ▸), was compared with 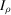 . In all models, when *V*_frac_ = 0.2, the *R*_p_ value of the minimum 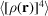 phase combination is larger than that of 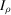 (Figs. 3 ▸ and S2). When *V*_frac_ = 0.7 for D-PS and D-CMCS and *V*_frac_ = 0.6 for P-PS, the *R*_p_ value for the minimum 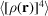 is larger than that based on 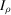 . Although there are conditions in which the *R*_p_ values are similar for both indicators, 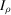 gives better results than 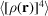 under several conditions. Both 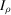 and 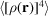 are indicators based on electron density: 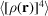 is calculated from the entire electron density, while 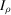 is calculated from the maximum and minimum electron densities. Therefore, 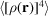 uses information from the intermediate-electron-density region, which is not used in 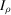 . This may cause the phase retrieval results of 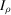 to be better under several conditions.

### 5.2. Phase retrieval for experimental data

The experimental data of LLC bicontinuous cubic phases acquired in previous X-ray single-crystal structure analyses (Oka, 2017 ▸; Oka *et al.*, 2018 ▸, 2020 ▸) were used as test data. In the calculation of the *R*_p_ value, the phase combinations previously derived from the model analysis were taken as the true combinations.

Monoolein, a type of lipid, and water form three type II bicontinuous cubic phases with TPMSs of G, D and P (Oka, 2017 ▸). The number of independent structure factors is from 8 to 14. 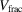 can be estimated to be around 0.43 to 0.54 (Table S2). The scatter plot shows that 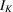 and 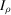 are roughly positively correlated, and that the *R*_p_ value is smaller toward the lower left [Fig. S3(*a*)]. This is the same as when 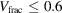 in the model densities. The phase combinations obtained from the structural models in the previous paper are also located at the lower left of the scatter plot. In the phase combinations with the minimum indicators, the calculated *R*_p_ values were almost zero (Table 1 ▸). This shows a good agreement between the structure shown by the indicator-based phase retrieval method and the structural model in the previous paper (Oka, 2017 ▸). For the two type II bicontinuous cubic phases of phytantriol with TPMSs of G and D, the number of independent reflections is 21, and the 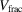 values are 0.57 and 0.66 (Oka *et al.*, 2018 ▸). These data are also similar to those of monoolein, and the structure shown by the phase retrieval method and the structure model in the previous paper (Oka *et al.*, 2018 ▸) are in good agreement (Table 1 ▸).

**Figure 5:**
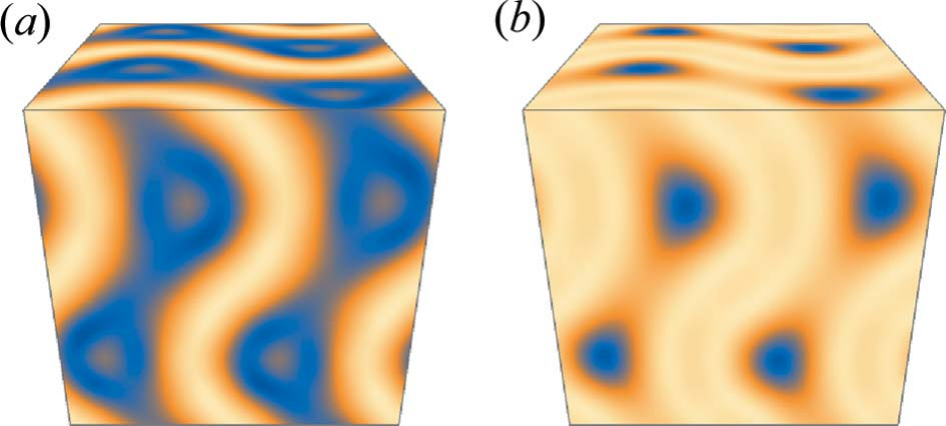
Electron densities of C_12_EO_6_ calculated with phase combinations of (*a*) minimum 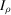 and (*b*) minimum 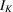 .

Hexa­ethyl­ene glycol monodo­decyl ether (C_12_EO_6_) and water form a type I bicontinuous cubic phase of G TPMS, with 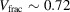 (Oka *et al.*, 2020 ▸). In the scatter plot of 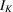 and 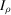 , the distribution of points is split in two on the lower left side [Fig. S3(*c*)]. 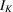 is a minimum near the point of the phase combination that was considered true in the previous paper (Oka *et al.*, 2020 ▸) ( 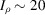 ), whereas 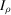 is not a minimum near the point. On the other hand, close to the point where 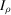 is minimum ( 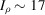 ), *R*_p_ is approximately 0.8 to 0.9. The corresponding electron densities are shown in Fig. 5 ▸. The electron density of the minimum *I_K_* is in very good agreement with the electron density in the previous paper (Oka *et al.*, 2020 ▸). A comparison of the electron density of the minimum *I_K_* [Fig. 5 ▸(*b*)] with Figs. 1 ▸(*b*) and 1 ▸(*c*) indicates that the width of the high-electron-density region on the TPMS side is wider, which is consistent with the large volume fraction on the TPMS side of C_12_EO_6_. With the minimum 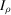 , the high-electron-density region on the TPMS side is narrower and the electron-density regions on the network sides are larger compared with the minimum *I_K_*. If the volume fraction is known in advance, then the electron density indicates that the phase combination with the minimum 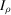 is not close to the true phase combination.

The fourth moment of the electron density 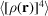 was also calculated in the experimental data, and the results are summarized in Table 1 ▸. The *R*_p_ values for the phase combination with the minimum indicator 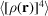 are comparable with those of 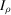 for four of the six experimental data sets. On the other hand, the *R*_p_ value of the minimum indicator 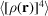 is approximately 0.1 for monoolein P and 0.2 for D, which are larger than that of 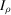 . For the difference of *R*_p_ ∼ 0.1, the electron densities show similar structures, but there are non-negligible differences in the details (Fig. S6). Considering the results with the model structures, *I*_*K*_ is the best indicator with the widest range of applicable conditions, followed by 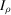 and then 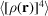 . Regardless of which indicator is used, care should be taken because there are volume fractions for which phase retrieval is difficult.

Unobservable structure factors cause truncation errors in the Fourier synthesis of electron density, 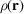 . The truncation errors may make the indicators inaccurate. However, given the observed amplitudes, the amplitudes of unobservable structure factors are probably less than 1% of the maximum amplitude. The amplitude decreases rapidly at high scattering angles due to the structural disorder of the liquid crystal (this is why the number of observable amplitudes is small). For this reason, the effect of the truncation error on the resultant structure model, *i.e.* indicators, seems insignificant. On the other hand, it may affect the phase retrieval of structure factors with weak amplitudes and cause the *R*_p_ value to deviate slightly from 0.

## 6. Conclusion

The indicators 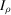 and 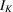 were determined to be useful for the crystallographic phase retrieval in LLC bicontinuous phases. The phase combination with the minimum indicator was very close to the true phase combination when the volume fraction was less than 0.6. Even for volume fractions of approximately 0.7 to 0.8, 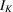 was effective to determine the proper phase combination as an indicator, although there were exceptions. In the scatter plots of 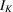 and 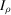 , when 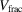 is small, the points extending to the lower left do not spread much [Fig. 3 ▸(*a*)]. On the other hand, when 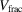 is large, the points in the lower left corner branch and spread out [Fig. 3 ▸(*b*)], which seems to be related to the certainty of the indicator.

The model used for the phase retrieval was a simple structure with different electron densities on the TPMS side and the network side. On the other hand, the sample of experimental data used for the phase retrieval has a more complicated structure. In the case of a type I bicontinuous cubic phase such as C_12_EO_6_, the polar region is located on the TPMS side and the nonpolar region is located on the network side. The hydro­phobic chain of the amphiphilic molecule is located in the nonpolar region, water is located in the TPMS in the polar region, and the hydro­philic part of the amphiphilic molecule is located in the polar–nonpolar interface side of the polar region. Therefore, the electron density differs among the three regions, with the hydro­philic part of the amphiphilic molecule having the highest electron density [Fig. 5 ▸(*b*)]. Phase retrieval was possible even in such a system.

It takes a long time to calculate the indicators of electron densities for all phase combinations; especially for 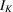 , it was necessary to calculate the eigenvalues of the Hessian matrix at each voxel, which takes a long time. On the other hand, the structures of the LLC bicontinuous cubic phase have highly symmetric space groups of 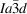 , 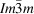 and 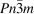 . Therefore, the computational time can be reduced by calculating only in the asymmetric units. In addition, the Babinet principle does not allow positive and negative electron densities to be distinguished in this paper; therefore, even when they are reversed, the number of combinations can be reduced. For an LLC bicontinuous cubic phase, the largest number of independent structure factors so far is 21 for phytantriol and C_12_EO_6–8_ (Oka *et al.*, 2018 ▸, 2020 ▸). When the number of voxels was 32^3^, the computation times of the Python script were 16 to 20 min using an AMD 3950X CPU with 16 cores and 32 threads. Calculation of 2^30^ phase combinations under the same conditions would thus take 10 to 14 days. It may be possible to increase the calculation speed by revising the algorithm. If the *I_K_* calculation is eliminated, then the calculation time will be much shorter.

Phase retrieval was performed using models and experimental data of LLC bicontinuous cubic phases. TPMS-like structures have been observed in thermotropic liquid crystals, polymers and other systems; it should also be possible to apply this phase retrieval method to these systems.

## Supplementary Material

Supporting information. DOI: 10.1107/S2053273322006970/ik5003sup1.pdf

## Figures and Tables

**Table 1:** *R*_p_ of the phase combinations with minimum indicator

Amphiphile	Type	TPMS	No. independent |*F*(**h**)|	*R*_p_ of minimum 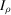	*R*_p_ of minimum 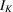	*R*_p_ of minimum 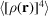
Monoolein (Oka, 2017 ▸)	II	P	12	0	0	0.109
		D	14	0.044	0	0.221
		G	8	0	0	0
Phytantriol (Oka *et al.*, 2018 ▸)	II	D	21	0.015	0.015	0.040
		G	21	0.033	0.017	0.017
C_12_EO_6_ (Oka *et al.*, 2020 ▸)	I	G	21	0.801	0.005	0.752
