# Supplementary material for: Crystallographic phase retrieval method for liquid crystal bicontinuous phases: indicator-based method
Source: Acta Crystallogr A Found Adv. 2022 Jul 28;78(Pt 5):430–6. doi: 10.1107/S2053273322006970 (PMC9434603; doi:10.1107/S2053273322006970)
Supplement: Supplementary file 1 [file a-78-00430-sup1.pdf]

FOUNDATIONS
ADVANCES

Volume 78 (2022)

Supporting information for article:

Crystallographic Phase Retrieval Method for Liquid Crystal Bicontinuous Phases: Indicator-Based Method

Toshihiko Oka

Crystallographic Phase Retrieval Method for Liquid Crystal Bicontinuous Phases : Indicator-Based Method

Supporting Information

Toshihiko Oka[1,2,*]

[1]Department of Physics, Faculty of Science and [2]Nanomaterials Research Division, Research Institute of Electronics, Shizuoka University, Shizuoka 422-8529, Japan

*oka.toshihiko@shizuoka.ac.jp

Contents

Table S1. Structure factors of models at different volume fractions.

Figure S1. Distributions of two indicators I_ρ and I_K of models at different volume fractions.

Figure S2-5. The R_p of the phase combinations with the minimum indicators in models.

Figure S6. Electron densities of monoolein P-TPMS calculated with phase combinations of minimum indicators.

Table S2. Volume fractions for the TPMS side and the network side.

Table S3. Constant distances from the TPMS to the polar-nonpolar interface in PS models.

Table S4. Indicator and Rp values in some phase-combinations of structure factors, monoolein.

Table S5. Indicator and Rp values in some phase-combinations of structure factors, phytantriol.

Table S6. Indicator and Rp values in some phase-combinations of structure factors, $C_{12}EO_6$.

Reference

Table S1. (a) Structure factors of G-PS model at different volume fractions.

G-PS							
			V_{frac}				
h	*k*	*l*	0.2	0.4	0.6	0.7	0.8
2	1	1	1.0000	1.0000	1.0000	1.0000	1.0000
2	2	0	0.6170	0.5946	0.5688	0.5590	0.5305
3	2	1	-0.0886	-0.0702	-0.0370	-0.0150	0.0147
4	0	0	-0.3056	-0.2357	-0.1036	-0.0199	0.0899
4	2	0	-0.2319	-0.1571	-0.0364	0.0363	0.1203
3	3	2	0.2857	0.1857	0.0249	-0.0751	-0.1823
4	2	2	0.1564	0.0924	-0.0003	-0.0550	-0.1097
4	3	1	0.1020	0.0585	-0.0068	-0.0445	-0.0781
5	2	1	-0.0300	-0.0140	0.0057	0.0122	0.0144
4	4	0	-0.0185	-0.0089	0.0034	0.0034	-0.0019
5	3	2	-0.0259	-0.0082	0.0087	0.0120	0.0079
6	1	1	-0.0602	-0.0192	0.0207	0.0274	0.0174
6	2	0	-0.0128	-0.0038	0.0053	0.0067	0.0044
5	4	1	-0.0318	-0.0080	0.0130	0.0145	0.0064
6	3	1	-0.0308	-0.0057	0.0132	0.0124	0.0009
4	4	4	0.0547	0.0083	-0.0260	-0.0230	0.0003
5	4	3	0.0372	0.0046	-0.0181	-0.0144	0.0037
6	4	0	-0.0211	-0.0019	0.0100	0.0070	-0.0036
5	5	2	0.0164	0.0009	-0.0082	-0.0050	0.0043
6	3	3	0.0092	0.0007	-0.0045	-0.0028	0.0028
7	2	1	0.0027	0.0002	-0.0012	-0.0007	0.0006
6	4	2	0.0079	0.0002	-0.0036	-0.0018	0.0029

Table S1. (b) Structure factors of G-CMCS model at different volume fractions.

G-CMCS							
			V_{frac}				
h	*k*	*l*	0.2	0.4	0.6	0.7	0.8
2	1	1	1.0000	1.0000	1.0000	1.0000	1.0000
2	2	0	0.4978	0.4629	0.4210	0.3944	0.3518
3	2	1	-0.1014	-0.0786	-0.0293	0.0106	0.0743
4	0	0	-0.3338	-0.2282	-0.0472	0.0744	0.2277
4	2	0	-0.2370	-0.1465	-0.0036	0.0787	0.1542
3	3	2	0.2721	0.1847	0.0422	-0.0504	-0.1540
4	2	2	0.1463	0.0912	0.0111	-0.0327	-0.0730
4	3	1	0.0917	0.0543	0.0083	-0.0135	-0.0308
5	2	1	-0.0323	-0.0100	0.0150	0.0219	0.0180
4	4	0	-0.0268	-0.0042	0.0259	0.0340	0.0252
5	3	2	-0.0256	-0.0062	0.0130	0.0146	0.0043
6	1	1	-0.0612	-0.0107	0.0282	0.0259	0.0019
6	2	0	-0.0118	-0.0029	0.0047	0.0056	0.0040
5	4	1	-0.0315	-0.0058	0.0145	0.0133	0.0006
6	3	1	-0.0303	-0.0022	0.0141	0.0084	-0.0088
4	4	4	0.0534	0.0143	-0.0229	-0.0266	-0.0099
5	4	3	0.0357	0.0077	-0.0146	-0.0151	-0.0041
6	4	0	-0.0206	-0.0010	0.0074	0.0039	-0.0036
5	5	2	0.0148	0.0031	-0.0043	-0.0047	-0.0038
6	3	3	0.0086	0.0021	-0.0024	-0.0034	-0.0030
7	2	1	0.0031	-0.0007	-0.0013	0.0003	0.0026
6	4	2	0.0069	0.0015	-0.0017	-0.0019	-0.0022

Table S1. (c) Structure factors of D-PS model at different volume fractions.

D-PS							
			V_{frac}				
h	*k*	*l*	0.2	0.4	0.6	0.7	0.8
1	1	0	1.0000	1.0000	1.0000	1.0000	1.0000
1	1	1	0.9041	0.8630	0.7804	0.7151	0.6144
2	0	0	-0.4306	-0.3649	-0.2420	-0.1454	-0.0103
2	1	1	0.2805	0.2163	0.1031	0.0242	-0.0762
2	2	0	0.2675	0.1759	0.0318	-0.0549	-0.1457
2	2	1	0.2609	0.1584	0.0065	-0.0784	-0.1531
3	1	0	-0.1550	-0.0837	0.0095	0.0481	0.0669
3	1	1	-0.0728	-0.0357	0.0085	0.0240	0.0271
2	2	2	0.1910	0.0861	-0.0424	-0.0924	-0.1091
3	2	1	0.0501	0.0178	-0.0163	-0.0246	-0.0190
4	0	0	0.0580	0.0150	-0.0200	-0.0216	-0.0047
3	2	2	0.0684	0.0146	-0.0295	-0.0301	-0.0082
3	3	0	0.0551	0.0095	-0.0232	-0.0203	0.0011
4	1	1	-0.0428	-0.0072	0.0169	0.0141	-0.0011
3	3	1	0.0521	0.0065	-0.0231	-0.0176	0.0050
4	2	0	-0.0366	-0.0031	0.0154	0.0096	-0.0052
4	2	1	-0.0245	-0.0010	0.0104	0.0053	-0.0047
3	3	2	0.0425	0.0001	-0.0191	-0.0083	0.0139
4	2	2	-0.0017	0.0001	0.0004	0.0001	0.0004

Table S1. (d) Structure factors of D-CMCS model at different volume fractions.

D-CMCS							
			V_{frac}				
h	*k*	*l*	0.2	0.4	0.6	0.7	0.8
1	1	0	1.0000	1.0000	1.0000	1.0000	1.0000
1	1	1	0.8111	0.7678	0.6574	0.5452	0.3220
2	0	0	-0.5267	-0.4341	-0.2631	-0.1172	0.1274
2	1	1	0.2662	0.2083	0.1067	0.0371	-0.0526
2	2	0	0.2581	0.1668	0.0331	-0.0300	-0.0520
2	2	1	0.2454	0.1527	0.0170	-0.0523	-0.0881
3	1	0	-0.1650	-0.0747	0.0385	0.0840	0.1001
3	1	1	-0.0786	-0.0286	0.0284	0.0447	0.0287
2	2	2	0.1767	0.0905	-0.0262	-0.0749	-0.0845
3	2	1	0.0472	0.0196	-0.0088	-0.0173	-0.0239
4	0	0	0.0600	0.0051	-0.0314	-0.0250	0.0109
3	2	2	0.0645	0.0192	-0.0218	-0.0269	-0.0135
3	3	0	0.0530	0.0075	-0.0179	-0.0091	0.0081
4	1	1	-0.0430	-0.0017	0.0208	0.0137	-0.0076
3	3	1	0.0498	0.0073	-0.0180	-0.0112	0.0031
4	2	0	-0.0367	0.0010	0.0161	0.0060	-0.0080
4	2	1	-0.0244	0.0023	0.0110	0.0009	-0.0126
3	3	2	0.0407	0.0040	-0.0170	-0.0102	0.0055
4	2	2	-0.0019	0.0023	0.0006	-0.0045	-0.0077

Table S1. (e) Structure factors of P-PS and P-CMCS model at different volume fractions.

P-PS							
			V_{frac}				
h	*k*	*l*	0.2	0.4	0.6	0.7	0.8
1	1	0	1.0000	1.0000	1.0000	1.0000	1.0000
2	0	0	0.9780	0.9222	0.8223	0.7480	0.6632
2	1	1	-0.6562	-0.5531	-0.3700	-0.2395	-0.0710
2	2	0	0.0472	0.0269	0.0019	-0.0083	-0.0064
3	1	0	-0.0920	-0.0660	-0.0238	0.0031	0.0357
2	2	2	0.3029	0.1907	0.0298	-0.0521	-0.1147
3	2	1	0.1306	0.0735	-0.0028	-0.0374	-0.0603
4	0	0	-0.1192	-0.0570	0.0155	0.0424	0.0562
3	3	0	-0.0826	-0.0345	0.0145	0.0265	0.0190
4	1	1	0.0940	0.0394	-0.0183	-0.0349	-0.0325
4	2	0	-0.0487	-0.0174	0.0110	0.0150	0.0081
3	3	2	-0.0694	-0.0210	0.0223	0.0272	0.0132
4	2	2	-0.0506	-0.0121	0.0178	0.0179	0.0047

P-CMCS				
			V_{frac}	
h	*k*	*l*	0.2	0.4
1	1	0	1.0000	1.0000
2	0	0	0.8184	0.6554
2	1	1	-0.6521	-0.5310
2	2	0	0.1085	0.0549
3	1	0	-0.0726	-0.0464
2	2	2	0.2929	0.1918
3	2	1	0.1191	0.0697
4	0	0	-0.1111	-0.0313
3	3	0	-0.0870	-0.0254
4	1	1	0.0895	0.0251
4	2	0	-0.0513	-0.0087
3	3	2	-0.0660	-0.0252
4	2	2	-0.0476	-0.0112

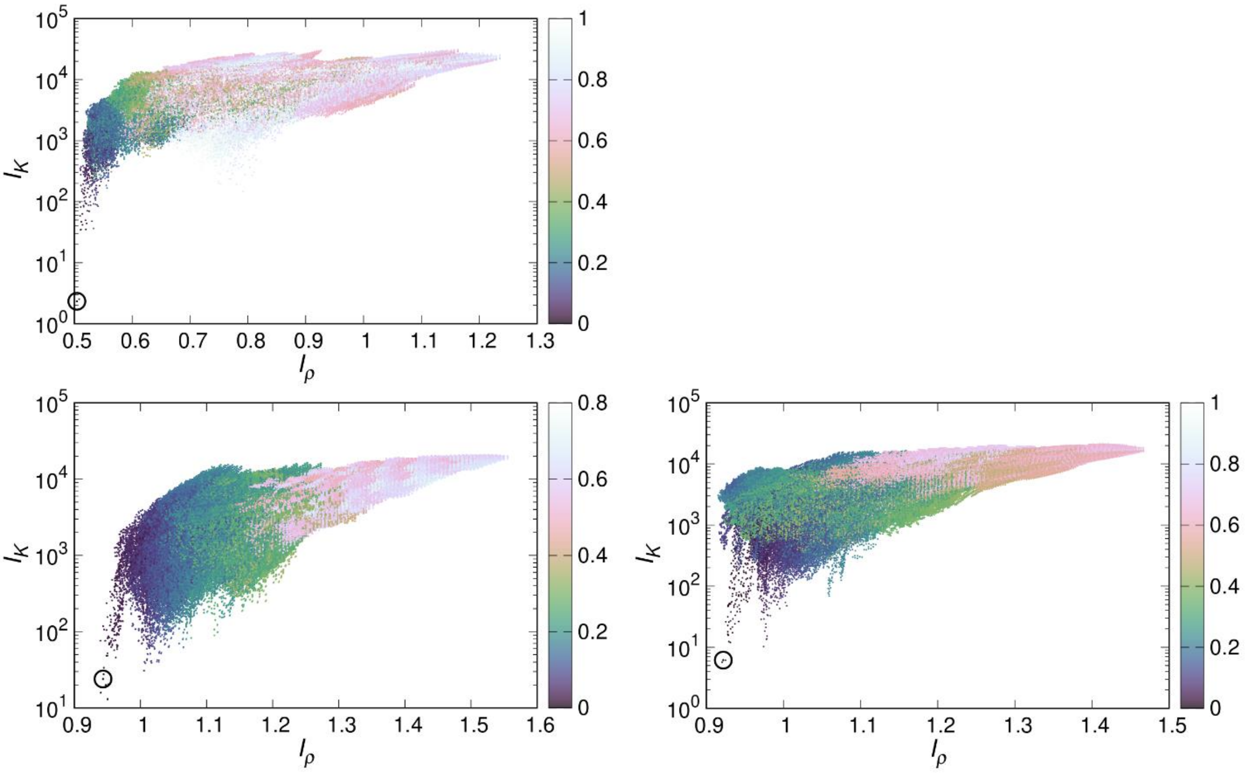

Figure S1. (a) Distributions of two indicators I_ρ and I_K of G-PS models at V_{frac}=0.2 (upper left), 0.6 (lower left), and 0.7 (lower right), respectively. The indicators were calculated from the electron densities using all phase combinations. The colors of the points indicate the R_{p} that compare the phase combinations with the true phase combination. The black circles show the true phase combination indicators of the model.

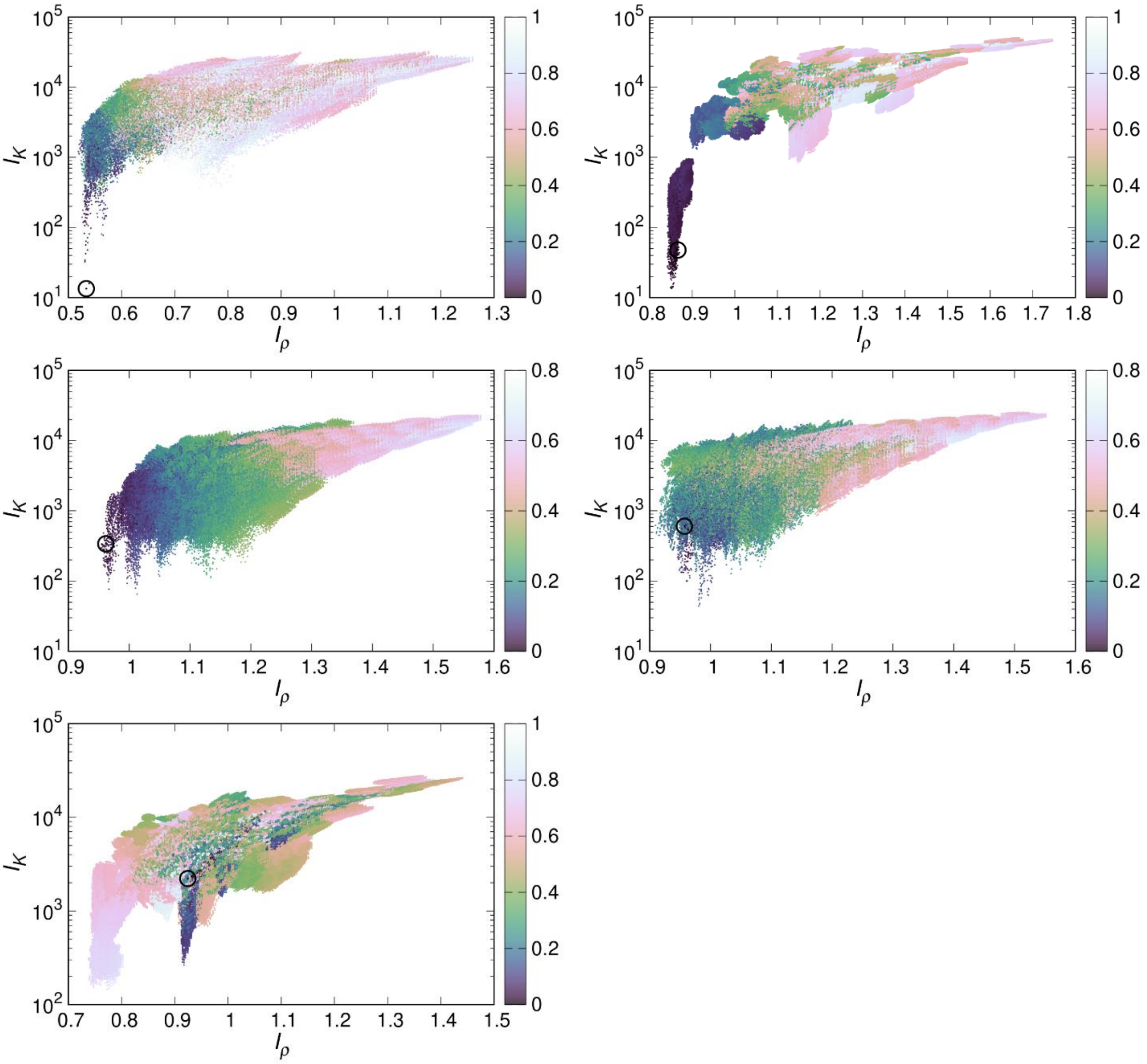

Figure S1. (b) Distributions of two indicators I_ρ and I_K of G-CMCS models at V_{frac}=0.2 (upper left), 0.4 (upper right), 0.6 (middle left), 0.7 (middle right), and 0.8 (bottom left), respectively. The indicators were calculated from the electron densities using all phase combinations. The colors of the points indicate the R_p that compare the phase combinations with the true phase combination. The black circles show the true phase combination indicators of the model.

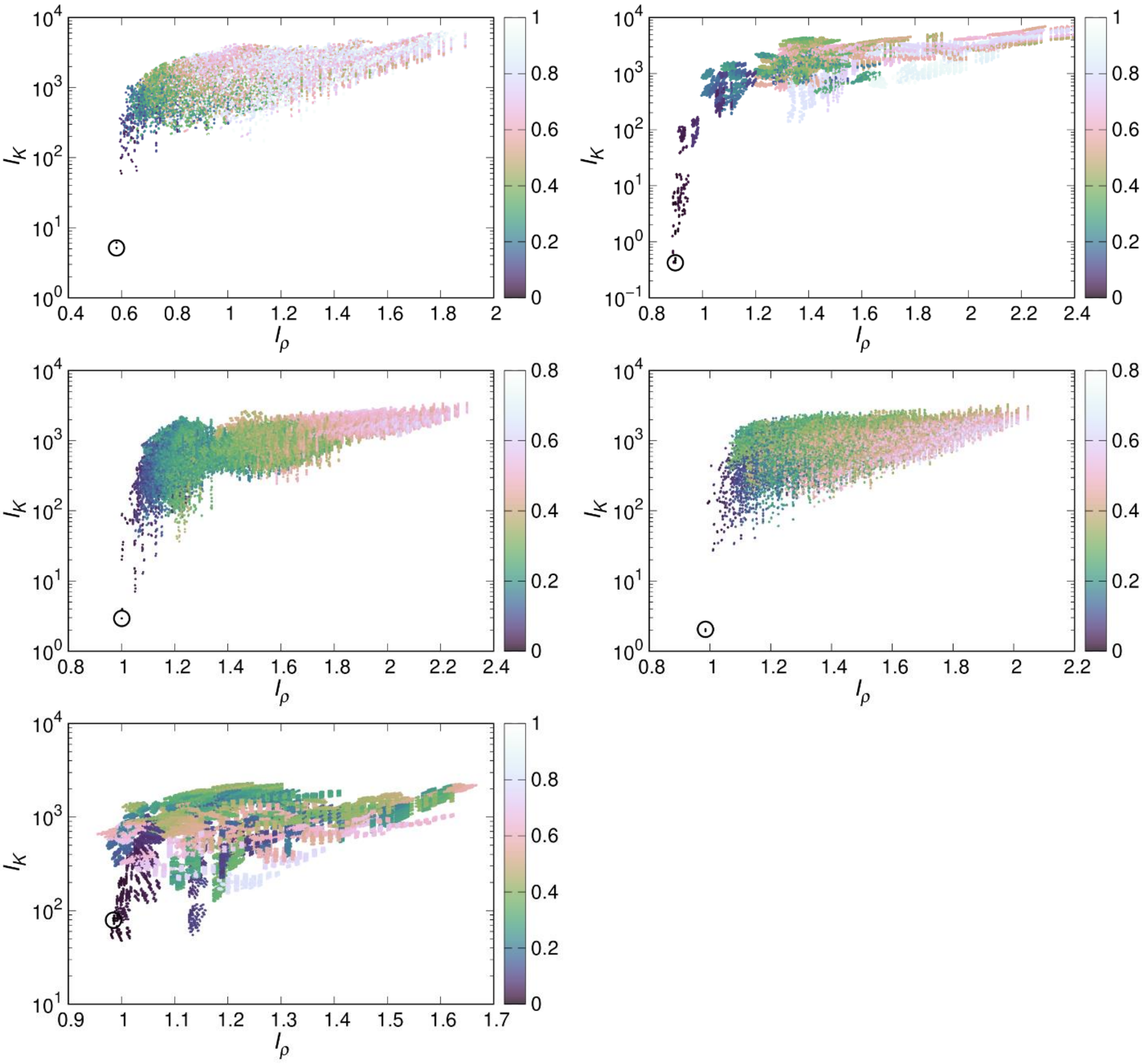

Figure S1. (c) Distributions of two indicators I_ρ and I_K of D-PS models at V_{frac}=0.2 (upper left), 0.4 (upper right), 0.6 (middle left), 0.7 (middle right), and 0.8 (bottom left), respectively. The indicators were calculated from the electron densities using all phase combinations. The colors of the points indicate the R_p that compare the phase combinations with the true phase combination. The black circles show the true phase combination indicators of the model.

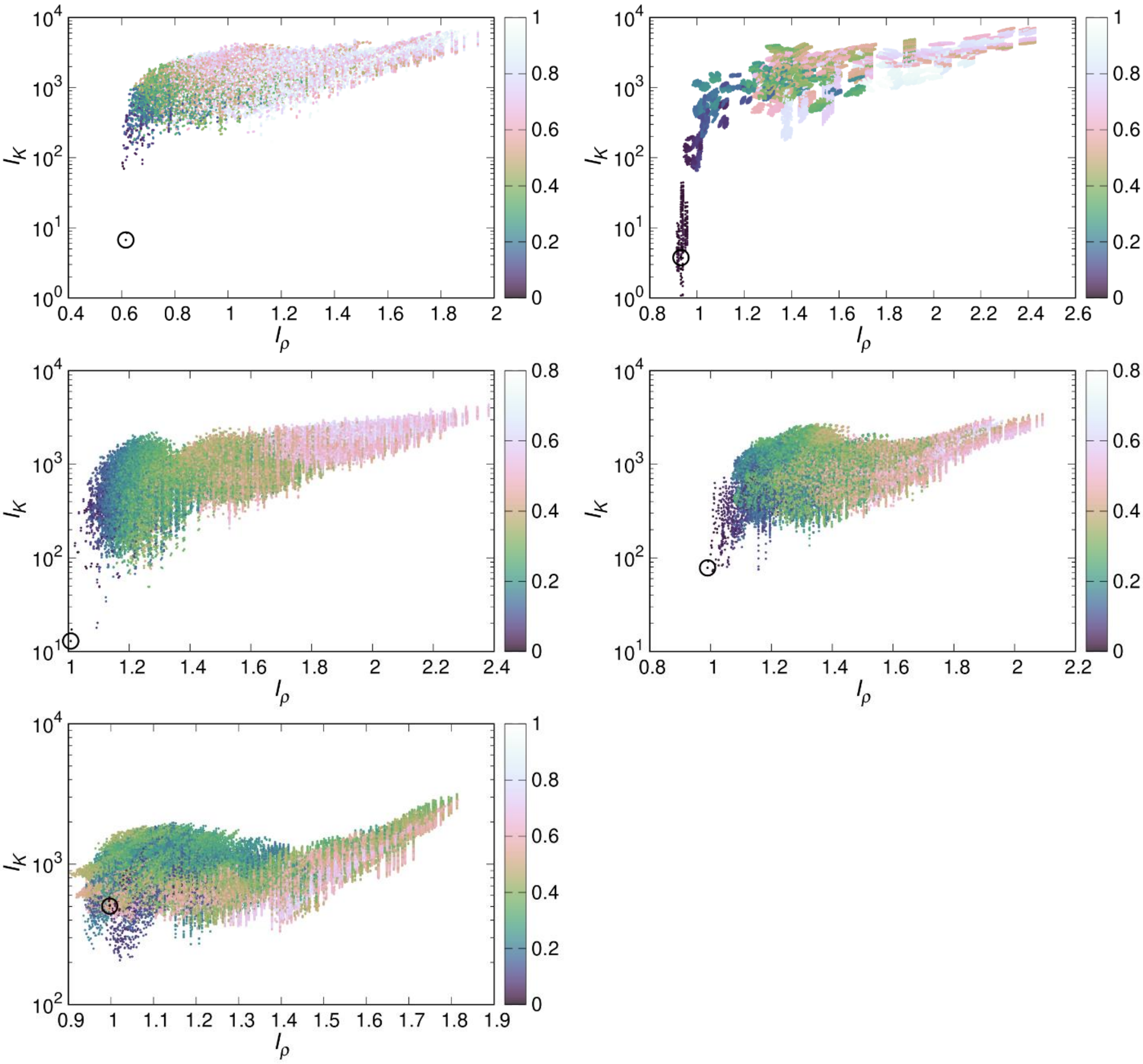

Figure S1. (d) Distributions of two indicators I_ρ and I_K of D-CMCS models at V_{frac}=0.2 (upper left), 0.4 (upper right), 0.6 (middle left), 0.7 (middle right), and 0.8 (bottom left), respectively. The indicators were calculated from the electron densities using all phase combinations. The colors of the points indicate the R_p that compare the phase combinations with the true phase combination. The black circles show the true phase combination indicators of the model.

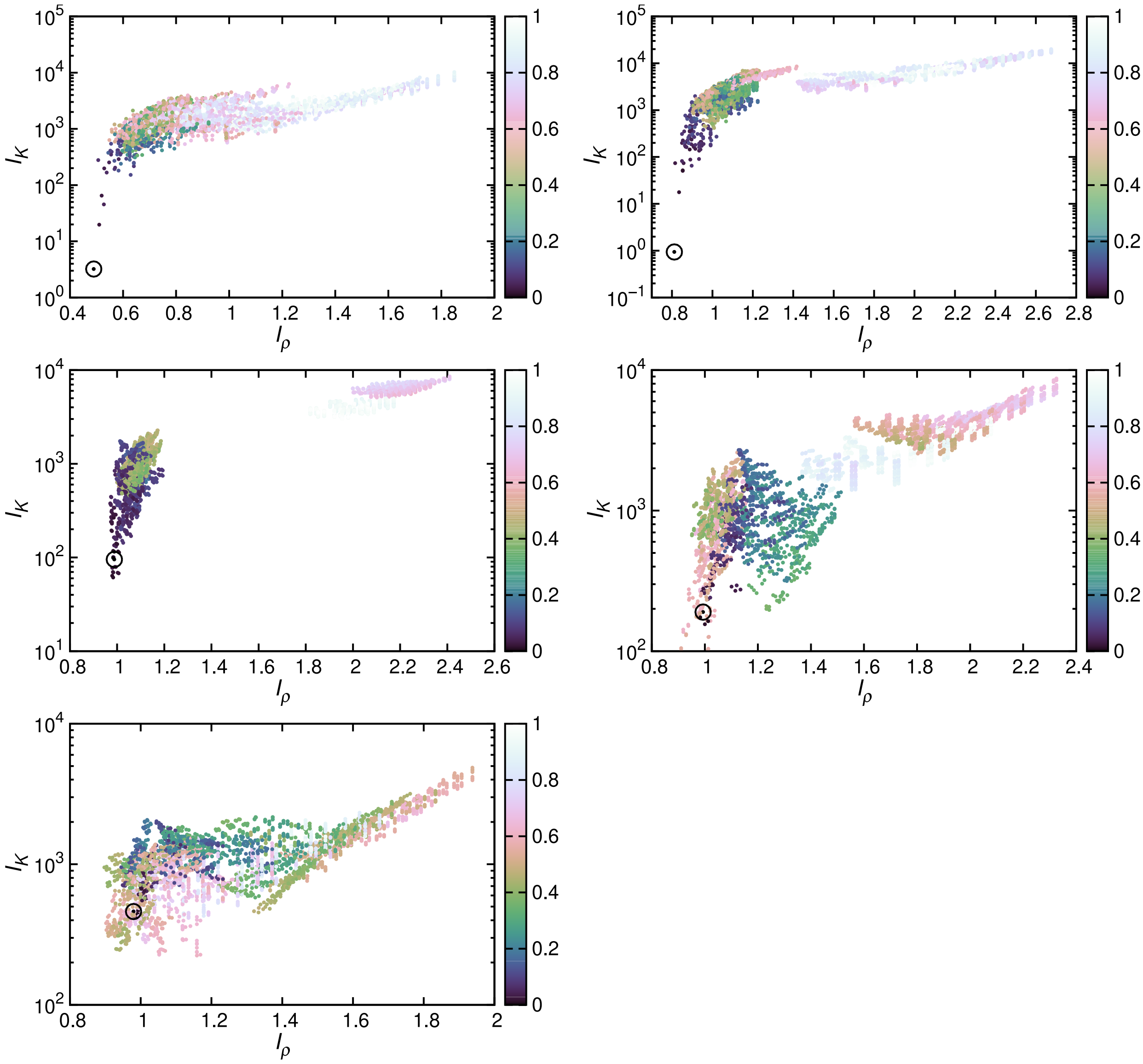

Figure S1. (e) Distributions of two indicators I_ρ and I_K of P-PS models at V_{frac}=0.2 (upper left), 0.4 (upper right), 0.6 (middle left), 0.7 (middle right), and 0.8 (bottom left), respectively. The indicators were calculated from the electron densities using all phase combinations. The colors of the points indicate the R_p that compare the phase combinations with the true phase combination. The black circles show the true phase combination indicators of the model.

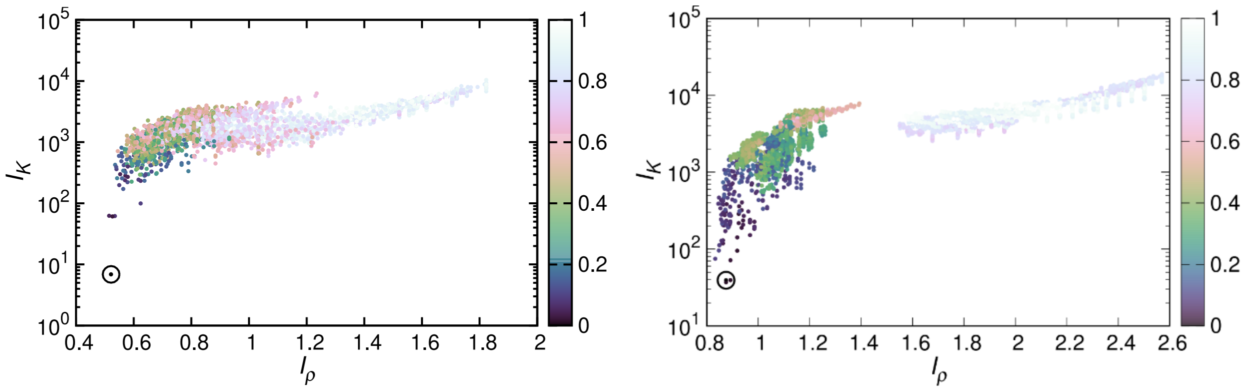

Figure S1. (f) Distributions of two indicators I_ρ and I_K of P-CMCS models at V_{frac}=0.2 (left), and 0.4 (right), respectively. The indicators were calculated from the electron densities using all phase combinations. The colors of the points indicate the R_p that compare the phase combinations with the true phase combination. The black circles show the true phase combination indicators of the model.

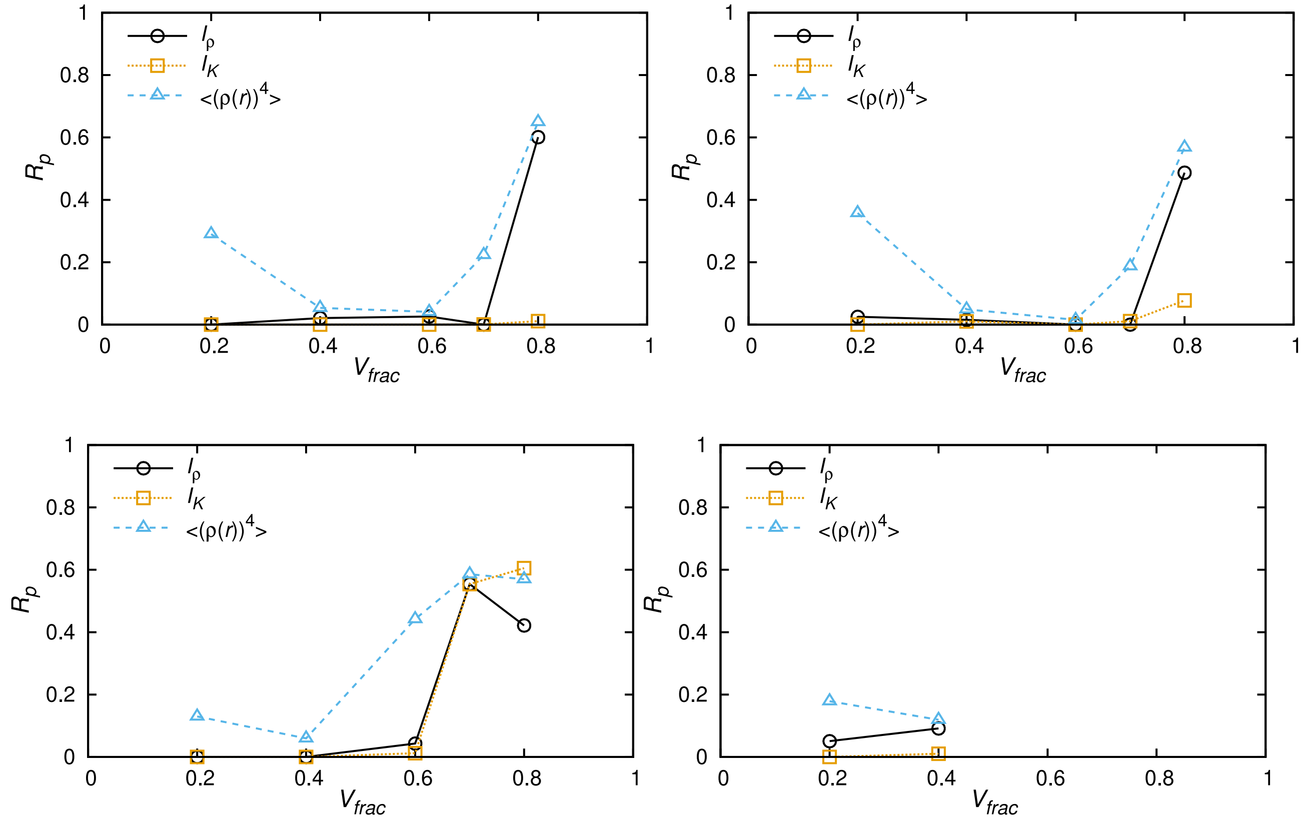

Figure S2. The R_p of the phase combinations with the minimum indicators in models based on D-PS (upper left), D-CMCS (upper right), P-PS (lower left) and P-CMCS (lower right) surfaces: I_ρ (black circle) and I_K (orange square), and $\langle(\rho(r))^4\rangle$ (sky blue triangle).

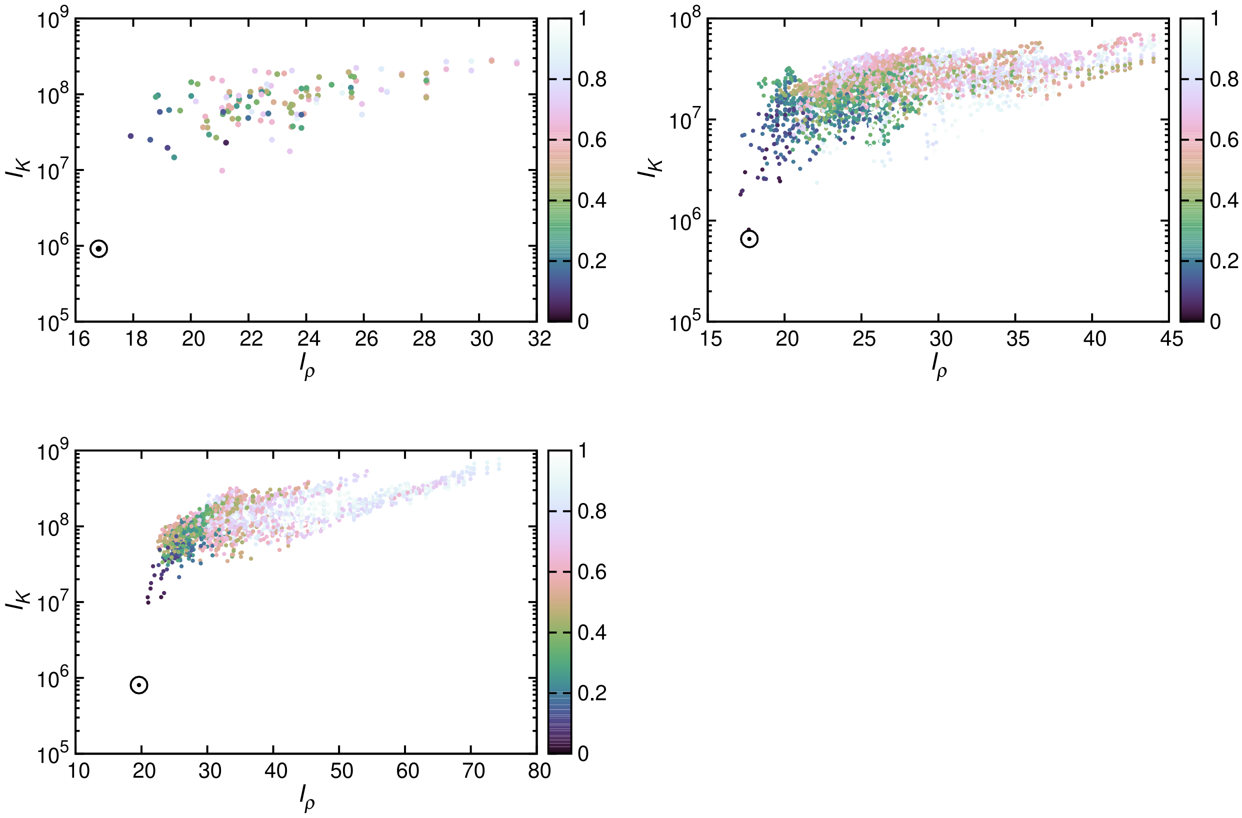

Figure S3. Distributions of two indicators I_ρ and I_K of monoolein whose structure similar to G (upper left), D (upper right), and P (bottom left) surface, respectively. The indicators were calculated from the electron densities using all phase combinations. The colors of the points indicate the R_p that compare the phase combinations with the determined phase combination in a previous paper[1]. The black circles show the indicators of the previously determined phase combination.

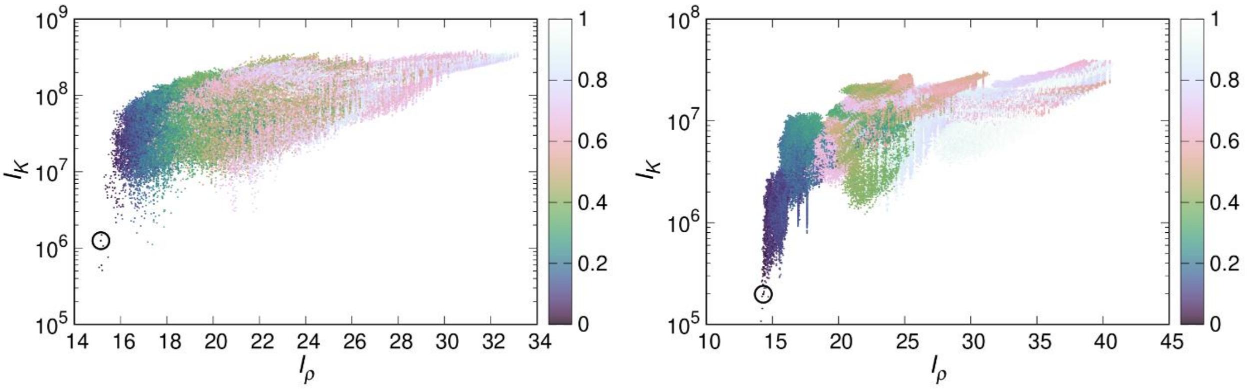

Figure S4. Distributions of two indicators I_ρ and I_K of phytantriol whose structure similar to G (left), and D (right) surface, respectively. The indicators were calculated from the electron densities using all phase combinations. The colors of the points indicate the R_p that compare the phase combinations with the determined phase combination in a previous paper[2]. The black circles show the indicators of the previously determined phase combination.

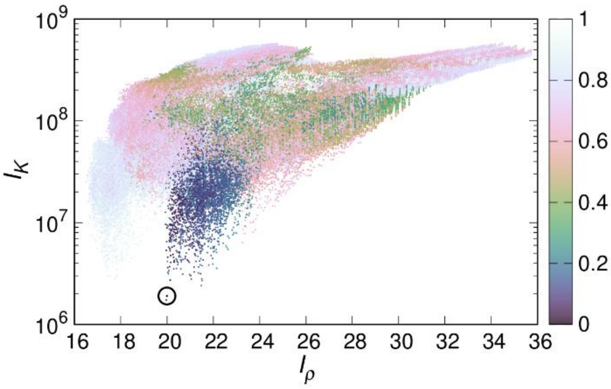

Figure S5. Distributions of two indicators I_ρ and I_K of $C_{12}EO_6$ whose structure similar to G surface. The indicators were calculated from the electron densities using all phase combinations. The colors of the points indicate the R_p that compare the phase combinations with the determined phase combination in a previous paper[3]. The black circle shows the indicators of the previously determined phase combination.

Figure S6. Electron densities of monoolein P-TPMS calculated with phase combinations of (a) minimum I_ρ and I_K, and (b) minimum $\langle(\rho(\boldsymbol{r}))^4\rangle$. The R_p values of (a) and (b) are 0. and 0.109, respectively.

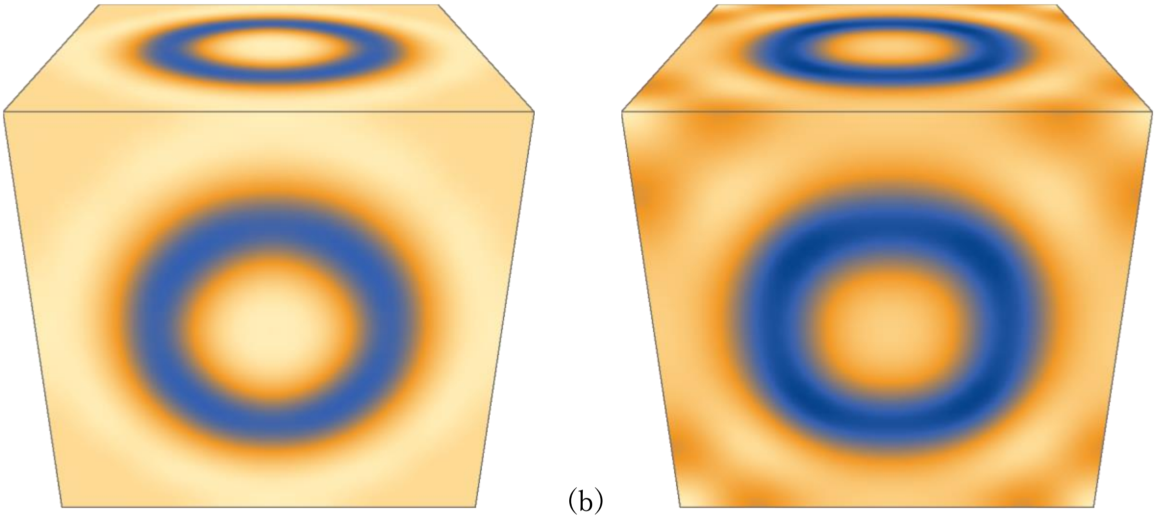

(a) (b)

Table S2. Volume fractions for the TPMS side and the network side. The volume fractions were calculated from the parameters described in previous papers.

amphiphile	LLC type	TPMS	TPMS side volume fraction		network side volume fraction	
			(water)	amphiphile	amphiphile	(water)
monoolein[1]	II	P	-	0.43*	0.13*	0.44
		D	-	0.44*	0.14*	0.42
		G	-	0.54*	0.16*	0.30
phytantriol[2]	II	D	-	0.574#	0.156#	0.271#
		G	-	0.657#	0.181#	0.162#
$C_{12}EO_6$[3]	I	G	0.209#	0.515#	0.276#	-

* PS model. monoolein length was fixed at 1.8 nm[1]. #PS model[2,3] .

Table S3. Constant distances from the TPMS to the polar-nonpolar interface in PS models.

volume fraction	0.2	0.4	0.6	0.7	0.8
G	0.0325387	0.0662825	0.102975	0.123424	0.146426
D	0.052425	0.106886	0.166386	0.199796	0.237792
P	0.0429265	0.0876974	0.137148	0.165427	0.198531

lattice constant is set as 1. The formula is described in the literature[4].

Table S4. Indicator and R_p values in some phase-combinations of structure factors, monoolein (a) P, (b) G, and (c) D.

(a) monoolein P					
h	k	l			
1	1	0	0	0	0
2	0	0	0	0	0
2	1	1	π	π	0
3	1	0	π	π	0
2	2	2	0	0	0
3	2	1	0	0	0
4	0	0	π	π	0
3	3	0	π	π	0
4	1	1	0	0	0
4	2	0	π	π	0
3	3	2	π	0	0
4	2	2	π	0	0
I_ρ			19.62*	22.61	74.26
I_K ($\times 10^5$)			8.050*	308.2	7745
$\langle(\rho(\boldsymbol{r}))^4\rangle$ ($\times 10^3$)			2.664	2.589*	54.88
R_p			0*	0.1091	0.9169

*minimum value among all phase combinations.

(b) monoolein G#					
h	k	l			
2	1	1	0	0	0
2	2	0	0	0	0
3	2	1	π	0	0
4	0	0	π	π	0
4	2	0	π	0	0
3	3	2	0	π	0
4	2	2	0	π	0
4	3	1	0	π	0
I_ρ			16.80*	21.09	23.84
I_K ($\times 10^5$)			9.171*	9.834	359.9
$\langle(\rho(\boldsymbol{r}))^4\rangle$ ($\times 10^3$)			1.472*	2.785	2.005
R_p			0*	0.6546	0.2907

*minimum value among all phase combinations.
#The phase combination at R_p=0 is the all π-shifted from that of the original data.

(c) monoolein D#						
h	k	l				
1	1	0	0	0	0	0
1	1	1	0	0	0	0
2	0	0	π	π	π	0
2	1	1	0	0	0	0
2	2	0	0	0	0	0
2	2	1	0	0	0	0
3	1	0	π	π	π	0
3	1	1	π	π	π	0
2	2	2	0	0	0	0
3	2	1	0	0	π	0
3	2	2	0	0	π	0
3	3	0	0	π	π	0
3	3	1	0	π	π	0
3	3	2	0	0	π	0
I_ρ			17.71	17.14*	18.65	26.54
I_K ($\times 10^5$)			6.593*	18.10	26.36	115.6
$\langle(\rho(\boldsymbol{r}))^4\rangle$ ($\times 10^3$)			1.391	1.355	1.262*	2.535
R_p			0*	0.0445	0.2206	0.2030

*minimum value among all phase combinations.
#The phase combination at R_p=0 is the all π-shifted from that of the original data.

Table S5. Indicator and R_p values in some phase-combinations of structure factors, phytantiol (a) D, (b) G.

(a) phytantriol D#						
h	*k*	*l*				
1	1	0	0	0	0	0
1	1	1	0	0	0	0
2	0	0	π	π	π	0
2	1	1	0	0	0	0
2	2	0	0	0	0	0
2	2	1	0	0	0	0
3	1	0	π	π	π	0
3	1	1	π	π	π	0
2	2	2	0	0	0	0
3	2	1	0	0	π	0
4	0	0	0	π	π	0
3	2	2	0	π	π	0
3	3	0	π	π	π	0
4	1	1	0	0	0	0
3	3	1	π	π	π	0
4	2	0	0	0	0	0
4	2	1	0	0	0	0
3	3	2	π	π	π	0
4	3	1	π	π	π	0
3	3	3	π	π	π	0
4	3	2	π	π	π	0
I_ρ			14.30	14.13*	14.55	24.46
I_K ($\times 10^5$)			1.981	1.075*	3.451	80.27
$\langle(\rho(\boldsymbol{r}))^4\rangle$ ($\times 10^3$)			0.9352	0.9319	0.9316*	1.842
R_p			0*	0.0145	0.0404	0.3299

*minimum value among all phase combinations.
#The phase combination at R_p=0 is the all π-shifted from that of the original data.

(b) phytantriol G#						
h	*k*	*l*				
2	1	1	0	0	0	0
2	2	0	0	0	0	0
3	2	1	π	π	π	0
4	0	0	π	π	π	0
4	2	0	π	π	π	0
3	3	2	0	0	0	0
4	2	2	0	0	0	0
4	3	1	0	0	0	0
5	2	1	0	π	π	0
4	4	0	0	π	0	0
5	3	2	0	0	0	0
6	1	1	0	0	0	0
6	2	0	0	π	0	0
5	4	1	0	0	0	0
6	3	1	0	0	0	0
4	4	4	π	π	π	0
5	4	3	π	π	π	0
6	4	0	0	0	0	0
5	5	2	π	π	π	0
6	3	3	π	π	π	0
6	4	2	π	π	π	0
I_ρ			15.15	15.07*	15.21	24.42
I_K ($\times 10^5$)			12.51	5.573	5.573*	426.7
$\langle(\rho(\boldsymbol{r}))^4\rangle$ ($\times 10^3$)			1.265	1.264	1.264*	1.868
R_p			0*	0.0328	0.0166	0.4154

*minimum value among all phase combinations.
#The phase combination at R_p=0 is the all π-shifted from that of the original data.

Table S6. Indicator and R_p values in some phase-combinations of structure factors, $C_{12}EO_6$.

$C_{12}EO_6$ G							
h	*k*	*l*					
2	1	1	0	0	0	0	0
2	2	0	0	0	0	0	0
3	2	1	0	0	π	π	0
4	0	0	0	0	π	π	0
4	2	0	0	0	π	π	0
3	3	2	π	π	0	0	0
4	2	2	π	π	0	0	0
4	3	1	π	π	0	0	0
5	2	1	0	0	π	π	0
4	4	0	0	0	π	0	0
5	3	2	0	0	0	0	0
6	1	1	0	0	0	0	0
6	2	0	0	π	π	π	0
5	4	1	0	0	π	0	0
6	3	1	0	0	0	0	0
4	4	4	π	π	π	π	0
5	4	3	π	π	π	π	0
6	4	0	0	0	0	0	0
5	5	2	π	π	0	π	0
6	3	3	π	π	π	π	0
6	4	2	π	π	π	π	0
I_ρ			20.00	19.96	16.52*	17.25	22.65
I_K $(\times 10^5)$			19.03	17.36*	253.4	203.5	476.3
$\langle(\rho(\boldsymbol{r}))^4\rangle$ $(\times 10^3)$			3.104	3.108	1.435	1.419*	1.857
R_p			0*	0.0049	0.8007	0.7521	0.5501

*minimum value among all phase combinations.

References

(1) Oka, T. Small-Angle X-Ray Crystallography on Single-Crystal Regions of Inverse Bicontinuous Cubic Phases: Lipid Bilayer Structures and Gaussian Curvature-Dependent Fluctuations. *J. Phys. Chem. B* **2017**, *121* (50), 11399–11409. https://doi.org/10.1021/acs.jpcb.7b08589.

(2) Oka, T.; Ohta, N.; Hyde, S. Polar–Nonpolar Interfaces of Inverse Bicontinuous Cubic Phases in Phytantriol/Water System Are Parallel to Triply Periodic Minimal Surfaces. *Langmuir* **2018**, *34* (50), 15462–15469. https://doi.org/10.1021/acs.langmuir.8b03320.

(3) Oka, T.; Ohta, N.; Hyde, S. T. Polar–Nonpolar Interfaces of Normal Bicontinuous Cubic Phases in Nonionic Surfactant/Water Systems Are Parallel to the Gyroid Surface. *Langmuir* **2020**, *36* (30), 8687–8694. https://doi.org/10.1021/acs.langmuir.0c00597.

(4) Qiu, H.; Caffrey, M. Lyotropic and Thermotropic Phase Behavior of Hydrated Monoacylglycerols: Structure Characterization of Monovaccenin [†]. *J. Phys. Chem. B* **1998**, *102* (24), 4819–4829. https://doi.org/10.1021/jp980553k.
